# Fatal Case of Rabies in a Captive White-Tailed Deer: A Case Report from Chiapas, Mexico

**DOI:** 10.3390/tropicalmed6030135

**Published:** 2021-07-16

**Authors:** Moisés Armides Franco-Molina, Silvia Elena Santana-Krímskaya, Baltazar Cortés-García, Jorge Alejandro Sánchez-Aldana-Pérez, Oscar García-Jiménez, Jorge Kawas

**Affiliations:** 1 Laboratorio de Inmunología y Virología, Facultad de Ciencias Biológicas, Universidad Autónoma de Nuevo León (UANL), 66455 San Nicolás de los Garza, Nuevo León, Mexico; silviasantana89@gmail.com; 2 Departamento de Rabia Paralítica y Garrapata, Inocuidad y Calidad Agroalimentaria, Servicio Nacional de Sanidad, 06100 Ciudad de México, Estado de México, Mexico; Baltasar.cortes@senasica.gob.mx; 3 Comité de Fomento y Protección Pecuaria del Estado de Chiapas, 29020 Tuxtla Gutiérrez, Chiapas, Mexico; jasap-25@hotmail.com (J.A.S.-A.-P.); senasica-dczpalenque@outlook.com (O.G.-J.); 4 Facultad de Agronomía, Universidad Autónoma de Nuevo León (UANL), 66054 General Escobedo, Nuevo León, Mexico; Jorge.kawas@mnademexico.com

**Keywords:** rabies, virus, bovine paralytic rabies, white-tailed deer, Mexico, Chiapas

## Abstract

Rabies is a fatal viral infection that causes enc ephalitis in warm-blooded animals, including humans. Dog-transmitted rabies is considered eradicated in Mexico; however, rabies is not being tested in livestock with neurological symptoms (one of the main manifestations of rabies disease). In this case report, we describe a rabies case in a white-tailed deer in the Santo Domingo ranch, in Catazajá, Chiapas, Mexico, where white-tailed deer are kept under captivity, and are meant for human consumption. This is the first report of a rabies case in white-tailed deer in Mexico. We also describe the challenges to obtain a rabies diagnosis and the lack of public health policies to ensure containment of the disease, as well as the lack of awareness among farmers in the area. One single confirmed case of rabies indicates that more animals are affected by the disease. The risk for human health and economical losses will remain unknown until rabies tests are routinely performed in animals that present neurological symptoms.

## 1. Introduction

Rabies is a global and fatal zoonosis that causes encephalitis in warm-blooded animals [1]. The causal agent is the rabies virus of the Lyssavirus genus of the Rhabdoviridae family [2]. The virus circulates primarily among domestic, feral, and wild animals, such as dogs, cats, monkeys, foxes, bats, raccoons, and skunks; it can easily be transmitted to humans via percutaneous bites or scratches [1].

Mass vaccination campaigns have drastically reduced dog-transmitted rabies [2]. In 2019, the World Health Organization declared Mexico as the first country that eliminated dog-transmitted rabies as a public health problem [3]. The common vampire bat (*Desmodus rotundus*) is now the leading vector for rabies in Latin America, and the most affected animals are cattle [2]. The disease presented by cattle due to the rabies virus is called bovine paralytic rabies (BPR) [2].

The Mexican states with tropical and subtropical climate (Chiapas, Hidalgo, Quintana Roo, San Luis Potosí, Tabasco, Veracruz, and Yucatán) are the most affected by BPR. Between 2007 and 2015, Mexico reported 1872 cases of BPR that amounted to a loss of USD 2.6 million per year for the cattle industry [2,4]. Despite the huge economical loses and the risk for human life, rabies remains a neglected disease [4,5]. Most cases of BPR are not officially reported due to the lack of specialized diagnostic laboratories and diffusion about the clinical symptoms related to this disease.

In Mexico, the white-tailed deer (*Odocoileus virginianus*) is kept as livestock for human feeding purposes. In the present case report, we describe a BPR case in white-tailed deer in the southern state of Chiapas. To our knowledge, this is the first report of a rabies case in white tailed deer livestock in Mexico.

## 2. Case Report

**Figure 1:**
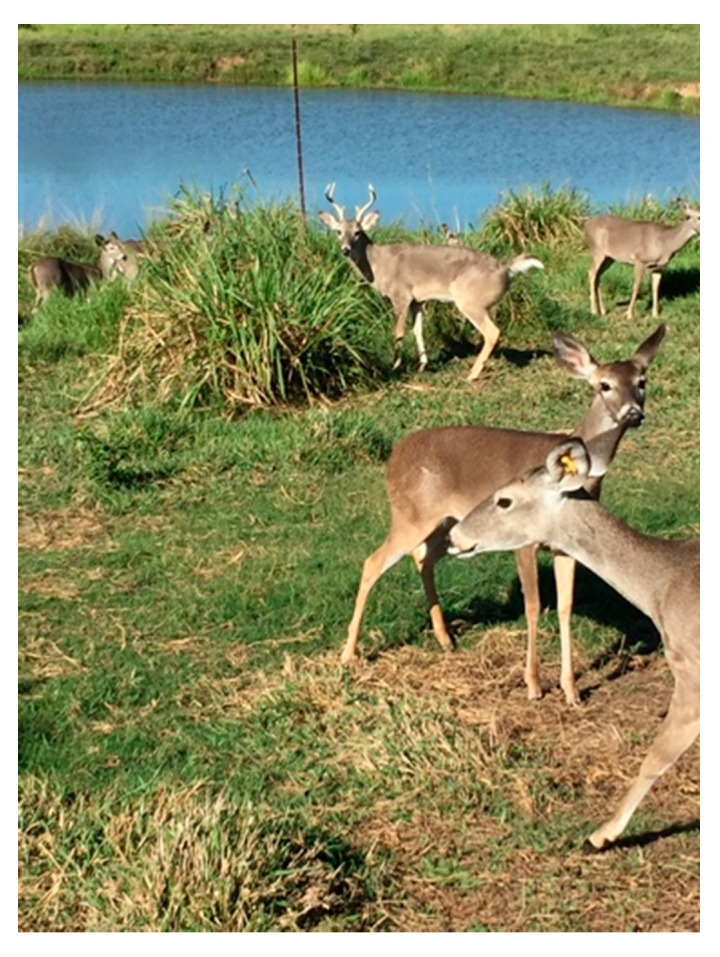
White-tailed deer grazing on *Sporobolus airoides* in the Santo Domingo Ranch.

The Santo Domingo Ranch, located in Catazajá, Chiapas, México, is a working ranch dedicated to the production bovine cattle and white-tailed deer (Figure 1). The deer diet consists of the commercial feed Trophy Maker (17% protein, macro and trace minerals and Vitamins), the Microfos VE-12 nutriment (macro and trace minerals, and A, D, E vitamins (MNA, Mexico), as well as ad libitum access to water and *Sporobolus airoides* for grazing.

**Figure 2:**
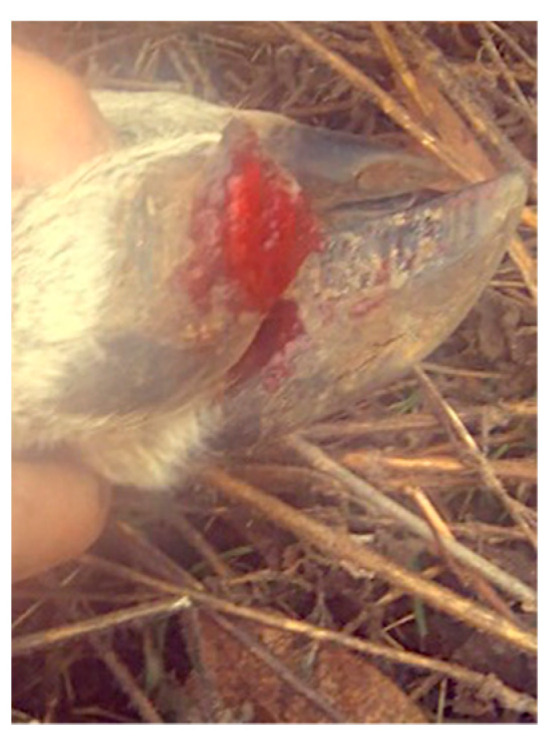
Pododermatitis in white-tailed deer 3 days before death.

In the month of October of 2020, seven deer died; this is considered a very short span of time for the deaths to be fortuitous. Furthermore, the deer that died presented similar symptomatology, which included hair loss, a tendency to limp, sudden prostration, and inability to escape from human proximity. The manifested symptoms were noticed for the first time two days before the first death. Some of the deer also presented pododermatitis (Figure 2). The pododermatitis lesions were treated with metamizole and trimethoprim-sulfamethoxazole, as well as local lavages with soap, water, and 5% copper sulfate (Supplementary Material Video S1). Before they died, the deer presented crystalline mucous running off from the nose, and lack of strength in the neck. The metamizole and trimethoprim–sulfamethoxazole treatment was administered again, and the animals that presented the symptoms were isolated in a room. Despite all measures, the deer that presented symptoms died.

In November, five more deer deaths occurred, and in December, three more deaths occurred, all with similar symptomatology. Table 1 depicts the deer symptomatology and deaths by month.

The local veterinary diagnosed all deaths as a probable intoxication with a venomous plant, which could have also caused the pododermatitis. Expert deer breeders from the north of the country were also consulted. Most of them agreed with the local veterinarian suggestion. Therefore, green areas of the ranch were cleaned-out from undesirable plants and muddy zones were disinfected with commercial lime (calcium oxide).

However, on 11 December 2020, one 4-month-old deer presented similar symptoms (prostration and neck bending), without pododermatitis or hair loss (Supplementary materials Video S2). Moreover, rabies was suspected by the ranch owner.

The deer was isolated in a room, where it subsequently died. The neck was immediately cut off, packed, sealed, and kept at 4 °C. The death was reported to the Unión Ganadera Regional De Catazajá, Centro de Salud Animal, Palenque, Chiapas, and to the Comisión México-Estados Unidos para la Prevención de la Fiebre Aftosa y otras Enfermedades Exóticas de los Animales (CPA). The corpse was necropsied at the ranch. All organs were found normal, and the presence of grass in the rumen was noted.

On 22 January 2021, the CPA reported a positive result for the rabies virus by direct immunofluorescence and a negative result for chronic wasting disease by ELISA-PrP. Rabies virus was evaluated in brain tissue, and chronic wasting disease in brain stem tissue. The CPA staff arrived at the ranch and vaccinated all the captive deer and cattle against rabies with the DERRI A PLUS vaccine (modified active rabies virus vaccine of vampiric origin Acatlán V-319 strain, obtained in BHK-21 C-13 cell tissue cultures) produced by Productora Nacional de Biológicos Veterinarios (PRONABIVE).

## 3. Discussion

Rabies remains a major neglected disease, it affects mainly poor and vulnerable populations living in areas with weak human and animal health infrastructures, and suspected rabies deaths are rarely reported [6].

Cases of rabies in deer have previously been reported in the United States of America [7], South Korea [8], Argentina [9], and China [10]. In most cases, the rabies in farmed animals correlates with wildlife reservoir, including bats, raccoons, and skunks. To our knowledge, this is the first report of rabies in white-tailed deer in Mexico, and the evidence presented in this case report seems to show that more cases are occurring in the southern state of Chiapas.

Despite the numerous deer deaths mentioned in this study, only one case was reported (the one positively diagnosed for rabies). It is worth mentioning that the undiagnosed deer deaths are only mentioned to accurately describe the situation that led to the rabies suspicion and diagnose.

The Santo Domingo ranch keeps bovine and deer livestock for feeding purposes. There are no documented cases of human rabies transmitted by the consumption of rabid animals’ meat; however, the World Health Organization (WHO) discourages eating meat from rabid animals because their handling and consumption are potential risks of contagion [11].

The bovine livestock from the ranch had been previously vaccinated against rabies and, unlike neighboring ranches from the zone, no bovine animal has presented the neurologic symptomatology. The deer had not been vaccinated against rabies when the cases occurred. They were vaccinated after the positive diagnosis with the DERRI A PLUS vaccine. After vaccination, symptoms and deaths among deer stopped.

Farmers from neighboring ranches have reported the death of cows that presented nervous symptomatology (personal communications), but the rabies tests were not performed. There are two possible reasons for the lack of testing for rabies. One reason is that farmers are not trained to properly preserve samples for rabies testing, and animal corpses have usually decomposed by the time CPA veterinaries arrive. The sample preservation is particularly challenging at the ranch conditions (the fresh frozen corpse was needed). Another reason might be that BPR is considered rare; therefore, farmers rather test for other casual agents, such as Histophilus somni, which can also cause nervous symptomatology and are easier to carry out. 

The pododermatitis is the only symptom that does not seem to relate with a rabies diagnosis. It is possible that the pododermatitis, which hinders mobility, makes deer an easier target for other animal bites, which is the main rabies transmission route [1]. 

According to the Mexican Official Norm (NOM-067-ZOO-2007), the common symptomatology of rabies in cattle includes aggressive behavior, salivation, prostration due to weakness in the posterior legs, and bending of the neck. The norm also states that rabies-like symptoms cannot be treated as a diagnosis given that different agents cause similar symptoms, and these symptoms can vary between animals; and positive cases have to be determined by authorized laboratories.

In the present report, a positive case for rabies was certified by the Comisión México-Estados Unidos para la Prevención de la Fiebre Aftosa y otras Enfermedades Exóticas de los Animales (CPA) laboratory. The diagnosis was carried out by a direct fluorescent antibody (DFA) test; which is also the standard test for rabies, post-mortem diagnose, approved by the CDC in the United States of America [12,13].

The positive rabies case in a mammal is also an indicator of an existing virus reservoir. In Mexico (and the rest of Latin America), the most common rabies reservoir is the vampire bath Desmodus rotundus [2]. We believe this might be the case; however, it would be necessary to capture baths from the area and screen for the presence of the virus to corroborate this. Furthermore, pododermatitis is the only symptom that does not seem to correlate with rabies; however, it decreases the deer mobility, leaving the animal more susceptible to bat bites.

In the case of the Santo Domingo Ranch, due to the rabies suspicion and diagnosis, only three farm workers directly handled the infected animal, and they were vaccinated to prevent rabies.

If rabies cases are not confirmed, the number of animals that are infected (and will die from rabies), as well as the economic damage caused by the disease, remains unknown. One confirmed case of rabies, however, is enough to know that there is a reservoir, and action must be taken. The diffusion of cases, such as the one reported, is important, so that ranchers become familiar with sample preservation, and report any suspected cases to the CPA for an accurate diagnosis.

Rabies remains a risk to public health, not only for farmers, but also for any other human that comes in direct contact with livestock; in rural zones, such as Catazajá, Chiapas, this includes most of the population. Therefore, it is necessary to establish mass vaccinations of livestock to prevent further economic losses and minimize risk to the human population.

## Figures and Tables

**Table 1:** Deer age, symptoms, and deaths by month.

Month	Animals Death (n°)	Age (Average)	Symptoms
October	7	4 (±1) years	Hair loss, sudden prostration, pododermatitis, neck bending, and death
November	5	4 (±1) years	Hair loss, sudden prostration, pododermatitis, neck bending, and death
December (1st week)	2	3 (±1) years	Hair loss, sudden prostration, pododermatitis, neck flexion, and death
December (2nd, 3rd, and 4th weeks)	1	4 (months)	Sudden prostration, neck bending, and death

## Data Availability

The data presented in this study are available upon request from the corresponding author.

## Supplementary Materials

The following are available online at https://www.mdpi.com/article/10.3390/tropicalmed6030135/s1, Video S1: Pododermatitis lesion being treated with 5% copper sulfate Video S2: Deer (4 months of age) presenting nervous symptomatology.
